# Data quality assessment in emergency medical services: an objective approach

**DOI:** 10.1186/s12873-023-00781-2

**Published:** 2023-01-30

**Authors:** Mehrnaz Mashoufi, Haleh Ayatollahi, Davoud Khorasani-Zavareh, Tahere Talebi Azad Boni

**Affiliations:** 1 Department of Health Information Management, School of Medicine, Ardabil University of Medical Sciences, Ardabil, Iran; 2 Health Management and Economics Research Center, Health Management Research Institute, Iran University of Medical Sciences, Tehran, 1996713883 Iran; 3 Department of Health Information Management, School of Health Management and Information Sciences, Iran University of Medical Sciences, Tehran, 1996713883 Iran; 4 Safety Promotion and Injury Prevention Research Center, Department of Health in Emergencies and Disasters, School of Public Health and Safety, Shahid Beheshti University of Medical Sciences, Tehran, Iran; 5 Social Determinants of Health Research Center, Saveh University of Medical Sciences, Saveh, Iran

**Keywords:** Emergency medical services, Data quality, Documentation, Assessment

## Abstract

**Background:**

In emergency medical services, high quality data are of great importance for patient care. Due to the unique nature of this type of services, the purpose of this study was to assess data quality in emergency medical services using an objective approach.

**Methods:**

This was a retrospective quantitative study conducted in 2019. The research sample included the emergency medical records of patients who referred to three emergency departments by the pre-hospital emergency care services (*n* = 384). Initially a checklist was designed based on the data elements of the triage form, pre-hospital emergency care form, and emergency medical records. Then, data completeness, accuracy and timeliness were assessed.

**Results:**

Data completeness in the triage form, pre-hospital emergency care form, and emergency medical records was 52.3%, 70% and 57.3%, respectively. Regarding data accuracy, most of the data elements were consistent. Measuring data timeliness showed that in some cases, paper-based ordering and computer-based data entry was not sequential.

**Conclusion:**

Data quality in emergency medical services was not satisfactory and there were some weaknesses in the documentation processes. The results of this study can inform the clinical and administrative staff to pay more attentions to these weaknesses and plan for data quality improvement.

## Introduction

Data quality is a complex topic thatincludes various dimensions such as accuracy, reliability, precision, completeness, timeliness, integrity and confidentiality [1, 2]. However, it is still a real challenge in many organizations including healthcare facilities [3]. In the healthcare industry, measuring data quality indicators are necessary to provide policymakers with reliable evidence for better decision-making and planning to deliver high quality healthcare services [4]. The quality of healthcare data is not only important for patient care, but also increases the effectiveness and efficiency of healthcare professionals and their services. High quality data should also be considered an essential prerequisite of information systems that are used to support healthcare services [5, 6].

Emergency department (ED) is one of the most important departments in a hospital, and a proportion of the patients, who refer to this department, are in critical conditions. In public health services, the performance of EDs is considered as a key benchmark for healthcare delivery, and meeting the key performance indicators such as providing high quality data in this department is regarded as one of the important issues for measuring the performance of the whole health system [7, 8]. In some studies, the use of electronic health records (EHR) has been suggested to measure performance of EDs, because the quality of electronic data is regularly evaluated and can provide managers with reliable reports [9–11].

On the other hand, the unique features of emergency care services, such as tasks complexity, high speed healthcare delivery, frequent transfer of care from one provider to another, multiple interruptions, high turn-over of patients and sometimes dealing with unknown or complex cases in the emergency environment, make this type of care prone to errors [12]. As a result, problems with data quality may occur more frequently in the ED than in other departments of the hospital and the process of data collection and quality assurance encounters a number of bottlenecks, which in turn, leads to unintended consequences [13]. However, the dynamic nature of emergency care services requires complete and accurate documentation of the care processes [14].

According to the literature, data quality in emergency medical services is not at a high level, and the needed data are not available at the point of need to the healthcare professionals and managers [15, 16] Therefore, it is necessary to assess the quality of data routinely to be able to improve it. The present study aimed to assess data quality in emergency medical services using an objective approach.

## Methods

This study was a retrospective quantitative study conducted in 2019 in which three dimensions of data quality; namely, completeness, accuracy and timeliness of data were assessed by reviewing different forms in the emergency medical records. Before conducting the research, ethics approval was obtained from the university ethics committee.

### Study setting

The study settings included three emergency departments located in three different teaching hospitals which were affiliated to three diverse medical universities. These emergency departments were the most crowded ones among other similar settings, and included triage, fast track, acute, and observation unites as well as a resuscitation room, an intensive care unit (ICU), and an ambulatory surgery room. The ED staff included emergency medicine specialists, general practitioners, nurses, and nurse assistants. Regarding documentation, a pre-hospital emergency care form was completed for patients who were visited by the pre-hospital emergency care services, and added to the patient emergency medical records which should be completed in the ED. Most of these forms were paper-based and few data elements including patient demographic data (mandatory fields) and para-clinical physician orders were also entered into the hospital information systems.

### Sampling

The research sample included the emergency medical records of patients who referred to the settings of the study through the pre-hospital emergency care services (*n* = 384). To reach this figure, we used Cochran’s sample size formula, where z-score was 1.96 (95% confidence interval), the standard deviation was (*p*= 0.5), and a margin of error was (d = 0.05) [17].

$$n=\frac{{\mathrm{z}}^{2}\mathrm{ p }\left(1-\mathrm{p}\right)}{\mathrm{d}2}$$

Then, the number of the patients, who were referred to the selected EDs by the pre-hospital emergency care services during the year before conducting the study, was identified and the sample size in each ED was calculated using stratified sampling method. Table 1 shows the proportion and the number of the records selected in each hospital.

**Table 1:** The proportion and the number of the records selected in each hospital

Hospitals	Frequency (%)	Number of the emergency medical records for review
Hospital A	8158 (22%)	84
Hospital B	14,619 (40%)	153
Hospital C	13,570 (38%)	147
Total pre-hospital referrals to the EDs	36,347 (100%)	384

To select the patient emergency medical records, one of the researchers (MM) attended the EDs, and records were selected randomly based on the unique patient identifiers.

### Data collection

A checklist was designed based on the data elements of the pre-hospital emergency care form, triage form, and emergency medical records, and approved by the research team before data collection. To assess data quality, three dimensions, namely completeness, accuracy, and timeliness were considered. In order to assess the completeness of data, emergency medical records and other forms were examined by one of the researchers (MM) to see what type of data had been documented. A subset of the records was also reviewed by another researcher (HA) to ensure agreement between the researchers.

To verify the data accuracy, common data elements in different data sources (emergency medical records, hospital information system and pre-hospital emergency care form) were identified by one of the researchers (MM), and then, their data were compared to identify any inconsistencies. In this comparison, the emergency medical records were regarded as the gold standard and the accuracy of other data sources was examined against this standard.

To examine data timeliness, the time of some procedures including paper-based physician ordering, data entry into the hospital information system, and receiving the results of diagnostic tests (laboratory tests, radiography, and CT scan) via hospital information system and picture archiving and communication system (PACS) were compared.

### Data analysis

Data were analyzed using descriptive statistics (frequency and percentage) and inferential statistics (Chi-squared test). In order to calculate data completeness, the number of documented data elements was divided by the total number of examined data elements and the percentage of completeness was reported for each data element. To measure data accuracy, the ratio of incorrect data elements was calculated via dividing the number of incorrect data by the total number of examined data elements [18]. To calculate the timeliness of data, the times of paper-based physician ordering, data entry into the hospital information system, and receiving the results of diagnostic tests via hospital information system and picture archiving and communication system (PACS) were compared.

## Results

In the following sections, the results of data quality assessment are reported for data completeness, data accuracy, and data timeliness, separately.

### Data completeness

In order to measure the level of data completeness, the data elements of the triage form, hospital admission and discharge form, and pre-hospital emergency care form were examined in terms of completeness or incompleteness. Table 2 shows the frequency of the data elements documented/not documented in the triage forms.

**Table 2:** Data elements documented/not documented in the triage forms

Status Data element		Documented Fr (%)	Not documented Fr (%)	Not applicable Fr (%)
Patient data	Name	384 (100)	0	0
	Surname	384 (100)	0	0
	Admission date	384 (100)	0	0
	Admission time	384 (100)	0	0
	Age	363 (94.5)	21 (5.5)	0
	Sex	365 (95)	19 (5)	0
	Pregnant	0	0	384 (100)
	Referral method	361 (94)	23 (6)	0
	Chief complaint	381 (99.2)	3 (0.8)	0
	Drug and food allergies	187 (48.7)	197 (51.3)	0
	Triage level	384 (100)	0	0
Life-threatening conditions	Level of consciousness	7 (1.8)	3 (0.8)	374 (97.4)
	Airway hazard	2 (0.5)	8 (2.1)	374 (97.4)
	Respiratory distress	3 (0.8)	7 (1.8)	374 (97.4)
	Cyanosis	3 (0.8)	7 (1.8)	374 (97.4)
	Shock symptoms	1 (0.3)	9 (2.3)	374 (97.4)
	Blood oxygen saturation level < 90	2 (0.5)	8 (2.1)	374 (97.4)
High-risk conditions of	Lethargy and drowsiness	2 (0.5)	34 (8.9)	348 (90.6)
	Severe pain or distress	3 (0.8)	33 (8.6)	348 (90.6)
	Medical history	80 (20.8)	304 (79.2)	0
	Medication history	60 (15.6)	324 (84.4)	0
Vital signs	Blood pressure	301 (78.4)	48 (12.5)	35 (9.1)
	Respiration rate	176 (45.8)	173 (45.1)	35 (9.1)
	Pulse rate	222 (57.8)	127 (33.1)	35 (9.1)
	Temperature	67 (17.5)	282 (73.4)	35 (9.1)
	Blood oxygen saturation level	245 (63.8)	104 (27.1)	35 (9.1)
Referral data	Referral ward	370 (96.4)	14 (3.6)	0
	Referral time	264 (68.8)	120 (31.2)	0
	Referral date	281 (73.2)	103 (26.8)	0
	The name of the triage nurse	366 (95.3)	18 (4.7)	0
	Triage nurse signature	341 (88.8)	43 (11.2)	0

As Table 2 shows, the demographic data were completely documented in most of the triage forms. The level of data completeness for chief complaints was (*n* = 381, 99.2%), and for drug and food allergies was (*n* = 187, 48.7%). Medical histories (*n* = 304, 79.2%) and medication histories (*n* = 324, 84.4%) were not completed in most cases. In total, the level of data completeness for the triage form by average was 52.3%.

In the pre-hospital emergency care form, more than 93% of the patients’ demographic data were completed. The time of departure from the patient location was not completed in any of the forms and the arrival time to, and the departure time from hospitals were only documented in 49 forms (12.8%). The level of data completeness for medication history, medical history, and drug allergies was (*n* = 318, 82.8%), (*n* = 338, 88%) and (*n* = 263, 28.5%), respectively. Overall, the level of data completeness for the pre-hospital emergency care form by average was 70%, and the completeness of the demographic data was more than the clinical data.

Regarding the emergency medical records, the level of data completeness for most of the demographic data was higher than 87%. However, the level of data completeness for the national identification number (*n* = 166, 43.2%), marital status (*n* = 230, 59.9%) and sex (*n* = 231, 60.2%) was less than other demographic data elements. In addition, the level of data completeness for final diagnosis, patient status at discharge, and post-discharge recommendations were (n = 277, 72.1%), (n = 133, 34.6%), and (n = 95, 24.7%), respectively. Among 384 emergency medical records, 13 were related to the fatal cases, and only for one case, the cause of death was reported. Overall, the level of data completeness for the emergency medical records by average was 57.3%, and the completeness of the demographic data was more than the clinical data. According to the results, the demographic data were more completed than the clinical data in all forms and across all three EDs.

## Data accuracy

To examine the level of data accuracy, data consistency among different data sources was measured. Data sources included emergency medical records (as a gold standard), pre-hospital emergency care form, and hospital information systems. Initially, the common data elements among these data sources were identified. To measure the level of data accuracy, the number of discrepancies between two data sources was divided by the number of common data elements. Initially, data consistency between the emergency medical records and hospital information systems was examined. However, the only common data elements between these two resources were demographic data which were consistent in 100% of cases. In fact, when a patient was admitted to the emergency department, the demographic data were entered into the hospital information system. Then, these data were printed and attached to the patient emergency medical records. Therefore, it was decided to examine the consistency of 6 common data elements in the pre-hospital emergency care form and emergency medical records (Table 3).

**Table 3:** Data consistency between the common data elements of the pre-hospital emergency care form and the emergency medical records

Pre-hospital emergency care form Emergency medical records		Documented Fr (%)	Not documented Fr (%)	Total Fr (%)	*P*-value
Chief complaint	Documented	353 (92.4)	2 (100)	355 (92.4)	> 0.05
	Not documented	29 (7.6)	0	29 (7.6)	
	Sum	382 (100)	2 (100)	384 (100)	
Final Diagnosis	Documented	133 (74.3)	144 (70.2)	277 (72.1)	> 0.05
	Not documented	46 (25.7)	61 (29.8)	107 (27.9)	
	Sum	179 (100)	205 (100)	384 (100)	
Drug allergies	Documented	74 (29.2)	32 (26.4)	106 (27.6)	> 0.05
	Not documented	189 (70.8)	89 (73.6)	278 (72.4)	
	Sum	253 (100)	121 (100)	384 (100)	
Medical history	Documented	183 (54.1)	17 (36.9)	200 (52.1)	0.029
	Not documented	155 (45.9)	29 (63.1)	184 (47.9)	
	Sum	338 (100)	46 (100)	384 (100)	
Medication history	Documented	163 (51.2)	26 (39.4)	189 (49.2)	> 0.05
	Not documented	155 (47.9)	40 (60.6)	195 (50.8)	
	Sum	318 (100)	66 (100)	384 (100)	
External cause of accident	Documented	91 (39.4)	0	91 (38.2)	0.000
	Not documented	140 (60. 6)	7 (100)	147 (61.8)	
	Sum	231 (100)	7 (100)	238 (100)	

As Table 3 shows, the highest consistency was related to the chief complaint (*n* = 353, 92.4%) and the lowest consistency belonged to reporting drug allergies (*n* = 74, 29.2%). In addition, there was no statistically significant difference between the data consistency of the chief complaint, final diagnosis, drug allergies and medication history. However, there was a statistically significant difference between reporting the external cause of accidents (*P*-value = 0.000) and medical history (*P*-value = 0.029) in these two data sources. It means that these two data elements were more documented by the pre-hospital emergency care staff compared to the ED staff.

## Data timeliness

To measure data timeliness, three sources of data; namely, emergency medical records, hospital information system (HIS), and picture archiving and communication system (PACS) were used to extract the time of some para-clinical procedures (laboratory, radiology and CT scan services) performed in the EDs (Table 4).

**Table 4:** Time intervals between the paper-based and computer-based documentation of diagnostic testing in the EDs

Type of services	Time intervals	Orders Fr (%)	Mean ± SD (minute)	Minimum (minute)	Maximum (minute)	Negative time interval Fr (%)
Laboratory	Time interval between the paper-based physician ordering and order entry into the HIS	220 (57.3)	24.2 ± 39.4	-53	277	13 (5.9)
	Time interval between order entry into the HIS and the accessibility of the results in the HIS	215 (56)	118.2 ± 66.2	-68	497	1 (0.5)
	Time interval between the paper-based physician ordering and the accessibility of the results in the HIS	214 (55.7)	144 ± 78.6	4	581	0
Radiography	Time interval between the paper-based physician ordering and order entry into the HIS	263 (68.5)	22.5 ± 35.4	-108	317	16 (6)
	Time interval between order entry into the HIS and the accessibility of the results in PACS	255 (66.4)	33.4 ± 39.6	-54	283	21 (8.2)
	Time interval between the paper-based physician ordering and the accessibility of the results in PACS	257 (66.9)	57.5 ± 53.9	-36	344	4 (1.6)
CT Scan	Time interval between the paper-based physician ordering and order entry into the HIS	178 (46.3)	28.7 ± 42.7	-40	306	9 (5)
	Time interval between order entry into the HIS and the accessibility of the results in PACS	173 (45.1)	26.2 ± 44.4	-85	195	31 (18)
	Time interval between the paper-based physician ordering and the accessibility of the results in PACS	173 (45.1)	54.3 ± 57.9	-52	297	19 (11)

Usually, the sequence of ordering and accessibility of the results should be as follows: paper-based physician ordering, order entry into the hospital information system (HIS), and the accessibility of the results in the HIS/PACS. After extracting the time documented for each procedure, the time interval between them was measured.

According to the results, there were positive and negative time intervals. The positive time interval indicated that the processes were performed sequentially; however, the negative time intervals indicated that the next process was performed before the previous one and they were not performed sequentially. As the results showed, in some cases the sequence of ordering and accessibility of the results was not maintained. As Table 4 shows, the highest mean value (144 ± 78.6) was related to the positive time interval between the paper-based physician laboratory ordering and the accessibility of the laboratory results in the HIS and the lowest mean value (22.5 ± 35.4) belonged to the time interval between the paper-based physician radiography ordering and order entry into the HIS. Moreover, most of the negative time intervals (*n* = 31, 18%) was related to the interval between the radiography order entry into the HIS and the accessibility of the results in PACS, which showed radiology orders were not documented in the HIS on a timely basis.

## Discussion

In this study, data quality was assessed in three emergency departments using an objective approach. To measure data quality, different data sources including a triage form, emergency medical records, pre-hospital emergency care form, hospital information systems, and picture archiving and communication systems were used, and data quality dimensions, which included data completeness, accuracy, and timeliness were assessed. As the results showed, the demographic data were more complete than the clinical data in three data sources; namely the triage form, emergency medical records, and pre-hospital emergency care form. Among the clinical data, the level of data completeness for some important data, such as life threatening conditions, drug allergies and vital signs (except blood pressure in the triage form) was not satisfactory, and among the above mentioned data sources, the level of data completeness in pre-hospital emergency care forms (70%) was higher than the other data sources.

It is noteworthy that in the present study and other similar ones, patient medical records were considered as a gold standard [19]. As a result, it is expected to see a higher level of data completeness in this data source. However, according to the results, the level of data completeness in the patient emergency medical records was 57.3% by average compared to the pre-hospital emergency care forms (70%). Although the completeness of the pre-hospital emergency care form was higher than the other data sources, according to Nisingizwe et al., if the completeness of data is less than 80%, it is considered poor [20]. The higher level of data completeness in the pre-hospital emergency care forms might be due to the importance of documenting each data element for a patient who is in a critical condition, strong dependency between documenting data in the pre-hospital care services and the continuity of care in the ED, and the importance of the legal issues.

The results are also consistent with the findings of other similar studies which showed that emergency medical records were usually incomplete and there were a number of missing data in the related electronic databases [14, 21]. This issue, in turn, can adversely affect quality of patient care [22]. The reasons for low quality documentation in the ED can be related to the high workload and low usage of digital infrastructure [23].

To measure data accuracy, the level of data consistency was examined in three different data sources. In fact, examining the level of data consistency is used to increase interoperability between different data sources [21, 24]. In addition, the best way to determine data accuracy is to determine the accuracy of data against another data source [20, 25, 26]. The results showed that most of the data elements were accurate (consistent), and there was no statistically significant difference between the data consistency in the emergency medical records and the pre-hospital emergency care form, except for the external cause of accidents and patient medical history which were more completed in the pre-hospital emergency care form. The importance of data consistency between different data sources, particularly in the emergency medical services has also been highlighted in other studies and it has been introduced as a predictor to determine the level of data completeness and accuracy [21, 27]. Understanding types and frequency of data errors in emergency care records helps healthcare organizations to develop practical strategies to improve data quality. Therefore, more attention should be paid to improve data consistency between emergency care data sources.

To measure data timeliness, the time interval between the paper-based physician ordering and order entry into the HIS, order entry into the HIS and the accessibility of the results in the HIS/PACS, and paper-based physician ordering and the accessibility of the results in the HIS/PACS were calculated and compared. The results showed that the time spent on the laboratory services was more than radiography and CT scan services, which can be related to the nature of the laboratory tests. However, there were some positive and negative time intervals. The negative time intervals showed that the sequence of procedures was not maintained. One of the reasons for this problem might be related to the complexity of the workflows in the ED. In addition, this might be due the ED workload, the severity of the patient condition and the urgency of providing some services which can interrupt routine workflows, priority of using verbal communications among the ED staff, lack of system integration, or paying less attention to the data documentation either in the paper-based or computer-based records [20]. Moreover, difficulties with continuous use of both paper-based and computer-based records can influence poor data quality [28]. As timely documentation of procedures is highly important in emergency care services, especially treatment, interventions, forensic issues, and quality assurance activities [29], process optimization should be taken into account to overcome current challenges.

Overall, it seems that data quality in emergency medical services was not satisfactory and there were some weaknesses in the documentation processes. Therefore, more practical strategies need to be developed to improve data quality. The use of health information technology in a wider scope can be one of these strategies that has been mentioned in several studies [29, 30]. Health information technology leads to improve data quality dimensions, such as completeness, accuracy, timeliness and accessibility by improving processes and facilitating clinical workflows. Moreover, providing emergency care staff with adequate training on documentation and conducting regular audits of data quality are other strategies which can help to improve data quality [31–33].

### Research implications

The nature of emergency care services makes it distinguished from other specialities. For example, in the ED, patients should be visited and managed as quickly as possible, and the accessibility of high quality data at the point of care can be extremely important for improving quality and speed of care [31]. While paying attention to data quality is of paramount importance for care delivery, experts recommend considering a distinction between the needed data for effective care delivery and documentation purposes. In addition, reducing the number of documented times, differentiating the data collection process for paper-based and computer-based records are other suggestions to improve data quality [34].

The results showed that medical and medication histories were not completed in many cases, while there is a perception that patient past history is important for creating a care plan [35]. Moreover, data entry into the HIS was not performed timely. These deficiencies might be due to different factors, such the characteristics of users, tasks, systems, environment, and the impact of technology [36, 37]. For example, task complexity in the ED may not be simply addressed by technology [31]. Therefore, more robust measurement and data collection guidelines are required to cover various workflows. This approach can improve data quality and allow for a more accurate assessment [34]. In addition, routine chart reviews in the hospitals, training healthcare staff in health data quality [30], providing constructive feedback, developing incentive mechanisms, and employing a data quality assurance team to establish mechanisms can also help to improve data quality in emergency care services [38].

### Research limitations

This study had some limitations. The first limitation was related to the limited number of data quality dimensions which were assessed. In fact, only three most important dimensions of data quality; namely, data completeness, accuracy, and timeliness were examined and other dimensions such as relevancy, compatibility and comparability were not assessed. Therefore, it is suggested to consider other data quality dimensions in the future research. Another limitation was related to the setting of the study which included only three EDs in three teaching hospitals. Although these three EDs had the highest number of annual visits, the quality of data can be different in other EDs, especially in other private and public hospitals. In this study, data quality was assessed retrospectively, as conducting a real-time observational study was not possible for the researchers. Moreover, as there was no national standard regarding the acceptable level of data quality in the ED, we were not able to compare the results against the standards.

In addition, although we investigated the level of data quality, the current documentation processes need further investigations to explore possible reasons for low level of data quality. Another limitation was related to choosing data elements to assess their timeliness. As the time of documentation was not available for many data elements, we focused on the para-clinical tests. This limitation can be addressed in the future studies by examining the timeliness of more data elements. Finally, we selected patient emergency medical records randomly, and we did not consider any specific patient condition. Therefore, there might be some data elements in the triage and pre-hospital emergency care form which were not completed, as they were not relevant to the patient condition, e.g. pregnancy for a child, and we consider them as “not applicable” rather than not documented.

## Conclusions

In this study, data quality was assessed in three emergency departments using an objective approach. The results showed that data quality in emergency medical services was not satisfactory. The demographic data had the highest level of completeness compared to the clinical data, and the pre-hospital emergency care form was more complete than other data sources. In addition, most of the examined data elements were accurate/ consistent. In terms of timeliness, the negative time intervals between some procedures suggested that there is a need to pay more attention to the sequence of para-clinical procedures in the EDs. These findings can be used to investigate bottlenecks and weaknesses in the documentation processes. Given that high quality data are necessary for providing high quality care, and a number of influencing factors may hinder proper documentation in emergency care services, it seems that providing a national minimum data set for emergency care services, training the emergency care staff in generating high quality data, and routine data quality audit can be useful for improving data quality. Further investigations are needed to use a combination of observational and qualitative research methodologies to identify bottlenecks in the process of emergency care documentation.

## Data Availability

The datasets used and/or analysed during the current study available from the corresponding author on reasonable request.

## Abbreviations

ED: Emergency Department
EHR: Electronic Health Records
HIS: Hospital Information System
PACS: Picture Archiving and Communication System
